# Treatise on the Oleum Jecoris Aselli, or Cod Liver Oil, as a Therapeutic Agent in Certain Forms of Gout, Rheumatism, and Scrofula; with Cases

**Published:** 1842-01

**Authors:** 


### Art. XI.

*Treatise on the Oleum Jecoris Aselli, or Cod Liver Oil, as a therapeutic agent in certain forms of Gout, Rheumatism, and Scrofula; with Cases.* By John Hughes Bennett, m.d., &c.—*London*, 1841. 8vo, pp. 180.

We are a little surprised that any one whose aim is the promotion of medical science, which we believe to be that of the author of this treatise, not mere personal notoriety, should take such pains in introducing to the notice of his brethren any *single* therapeutic agent; how useful soever that agent may have appeared to him. We think that from so respectable an authority as Dr. Bennett, a simple intimation in any of the journals that he had found cod-liver oil useful in such and such cases, and had witnessed its successful employment in Germany, would have secured all practical ends. The author has not even the excuse of having to deal with an agent of delicate use or dangerous application, in regard to which minute details might be justifiable. On the contrary, from Dr. Bennett's own showing, cod-liver oil belongs to a class of remedies of such equivocal powers, as might have almost justified the use of the term "dietetic" instead of "therapeutic" in the title-page of his book. At p. 42, he tells us that "The Laplanders, and the inhabitants of some northern nations, take it habitually, and consider it a delicacy. Even in Shetland (he says), I am informed by Dr. Edmonstone, that it is used extensively, when fresh, as an article of diet, instead of butter, and that it is universally considered both palatable and wholesome." At p. 61, we are told that Brefeld has seen the peasants swallow from half a pint to a pint of it, at a time. Surely an article which may be used so freely and continuously may claim a dietetic reputation.

In an Historical Introduction, Dr. Bennett informs us that the oil has been used immemorially in some of the northern nations as a cure for rheumatism. In Holland, it had long been employed by the vulgar in rachitic cases, before its virtues had been recognized by medical men. Dr. Bennett has heard that it is still employed in Scotland in rheumatic cases. Dr. Kay, of Manchester, employed it with success in the Infirmary of Manchester in 1789; and Dr. Bardsley previously to 1807. Dr. Shenk's papers in Hufeland's Journal, which were published in 1822 and 1826, led to its general use in Germany. And of late years it has been employed in Belgium and France, and partially in this country.

In the first section, in which "The natural and commercial history of cod-liver oil" is narrated, there is exceedingly little to detain us. We are informed that the oil is obtained from several species of gadus. The various processes of procuring the oil are detailed; but Dr. Bennett is not prepared to prefer any particular one. (p. 27.)

The second section treats of "The physical and chemical properties" of the oil. The purified sort obtained by Mr. Donovan, who has experimented for this purpose, has a specific gravity 0·934; is of a pale yellow colour, and the taste resembles "that of a cod boiled for the table, when in excellent condition." We shall by and by see that this is a much too favorable account of its saporous qualities; and we may here observe that the darker coloured oil is more richly replenished than the lighter, with that peculiar principle or ingredient, to which, as will presently appear, cod-liver oil owes such therapeutic properties as it possesses.

The results, in a tabular form, of one or two chemical analyses of the oil, are given at pp. 31-2-3; connected with which the most material and practical point is, that the oil has been found to contain iodine. Its therapeutic action, we are told, led Dr. Kopp to suspect the presence of iodine; and his shrewd conjecture was verified by De l'Orme. The results of two trials are stated, from which it appears that in one 0·324, in the other 0·162 per cent. of iodine was found in 100 parts of oil.

In section third, the "Action of cod-liver oil on the human economy" is described. Its sensible effects seem vague or null. Thus it is mentioned (p. 45) that "some practitioners have noticed an increased number of stools to follow its employment; but Brefeld states that although diarrhœa may occur during the use of the oil . . . . . the oil is not the cause of it." Professor Romberg has found it act as an astringent. Dr. Bennett endeavours to reconcile these apparent discrepancies by remarking that diarrhœa may depend in different cases on different causes.

The following were the effects of the administration of the oil in seventy-one cases:

"*Stomach.* Nausea was observed in three cases; vomiting, also, in three cases; in one case loss of appetite, and sensation of ardor in the stomach; the voracious appetite often observed in rachitic children was diminished; in one case cure of fetid breath.

"*Intestinal canal.* A greater or less increase in the alvine evacuation in seventeen cases.

"*Urinary apparatus.* Cure of incontinence of urine in two cases; acceleration of the urinary secretion, with a bricky sediment, in eight cases.

"*Generative apparatus.* Increase of the menstrual evacuation, so strong as to render a suspension of the oil necessary. The same phenomenon was observed to repeat itself several times on the oil being again given; in one case re-establishment of the menses.

"*Integumentary system.* Increased diaphoresis in twelve cases; in one of these the perspiration was only observed on the inferior extremities; in two others it had the odour of the oil; in three cases it was preceded by a heat over the whole body; in another the body was cold; in one case there was a burning tingling of the skin; in two cases an eruption of small red spots with itching; in the last cases a cure was not obtained.

"In a great number of cases the pains were more or less increased by the first doses of the medicine." (p. 47.)

If we add together the cases in which the effects are stated, they appear to amount to forty-three. Thus the result in a considerable proportion of the seventy-one cases may be presumed to have been either indeterminate or unsatisfactory; the oil either proving totally inert, or else causing symptoms which led to its early suspension.

It is Dr. Bennett's opinion that it is to the iodine that the oil owes its efficacy in scrofula. In reply to the objection that the oil contains only $\frac{1}{4000}$ part of this substance, as Falker asserts, and that therefore the presence of iodine in so minute a quantity cannot account for the curative properties of the oil, Dr. Bennett urges among other things that "iodine thus occurring in a natural production may be supposed to possess a stronger therapeutic action than when obtained by chemical means," and (p. 52) Falker ascribes the therapeutic efficacy of the oil to its gum resin.

Pages 53-60 inclusive are occupied with an exposition of the views of Dr. Ascherson, of Berlin, as regards the morphology of scrofula, and the therapeutic action of the oil in correcting the strumous diathesis. These views are certainly not devoid of ingenuity and intellect, although, as yet, they must be regarded as wholly or in great part hypothetical, and requiring ample verification.

As regards the doses of the oil and the length of time during which it must be employed, we find that it is best given in quantities of from half an ounce to four ounces, either separately or in one dose, daily. Its use must be continued "a month at least, often six months, and sometimes for years." (p. 50.)

It is always an advantage both to patient and physician, that a drug should be as agreeable as possible. Dr. Bennett would persuade us that the character of the oil, in this respect, is not so bad as has been represented; but his own very equivocal comparison will, with many, establish the very opposite conclusion to that he intended. "For my part," he remarks, "I do not think it more disagreeable to take than castor oil"! Dr. Rust ominously directs (p. 62), that "when the patient swallows the oil, he should shut his eyes, and compress both nostrils with his fingers;" and Brefeld observes (p. 63) that the oil "is certainly not calculated for luxurious or imaginative females." "One medical gentleman of eminence in the profession informed me," says Dr. Bennett, "that he would rather die than take the first dose." (p. 64.) Nevertheless, if the oil be really very useful, its odorous and saporous qualities, however atrocious, will not be an insuperable objection: for we agree with Brefeld that "when it is necessary, the patient will little trouble himself about what will do him good," (p. 63,) and with Dr. Bennett's simply expressed belief that "it is probable that the majority of my countrymen, as well as individuals [!] on the continent will swallow any substance which holds out the hopes of cure, after having suffered a few years from protracted rheumatism or gout."

The diseases which, it is asserted, oleum jecoris aselli is capable of curing are various and grave. These are, rheumatism and gout; scrofula; rachitis; malacosteon; scrofulous caries of the bones; atrophia mesenterica; phthisis pulmonalis; chronic skin diseases; scrofulous ulcers; scrofulous diseases of the eye; and other scrofulous diseases. It is but justice to Dr. Bennett to state that he seems (p. 48) to attribute the success of the oil in most or in all of these cases to the power of the iodine in correcting the scrofulous diathesis, the connexion with and dependence of these "cachectic diseases" with scrofula being assumed. And hence he accounts (p. 48) for the oil having produced good effects in a variety of cases which at first appear opposite to each other. We have also to add, that Dr. Bennett advises an accurate diagnosis as to whether the case be really of a nature proper for treatment by the cod-liver oil; since this substance, though effectual against rheumatism and scrofula, and also, as Dr. Bennett thinks, against gout, (which Brefeld denies,) is inert against neuralgia, an affection which may, in some cases, be confounded with rheumatism or gout. Care must therefore be taken that the oil may not be unjustly charged with failure, from being mistakenly applied to cases not really such as it is adapted and designed for.

Following the author's own plan, we now pass to the cases, respecting which we would make one general observation: namely, that Dr. Bennett's clinical notices of the particular operation of the oil, are in the greatest degree meager and vague. In the first place, scarcely in one instance are the state and appearance of the excretions either previously or subsequently to the administration of the oil recorded; nor what effect, if any, the oil had upon these. The effects of the medicine are, in the majority of the cases, stated in the same unsatisfactory, we might say, unprofessional style, as the following, (p. 81:) "Some bottles of the oil cured the girl so completely that not a trace of the affection remained, and it has never returned." This deficiency we regard as deducting greatly from the value and practical utility of Dr. Bennett's cases. It is not our intention to go over these cases. There is nothing in Dr. Bennett's mode of detailing them, to make it useful or necessary to do so. While the reader of our pages will lose nothing by this omission, Dr. Bennett will probably think that we fulfil the principal object when we state that radical cure or palpable amelioration, even in the serious cases already enumerated, evinced the therapeutic efficacy of cod-liver oil. We shall not curiously enquire how far the improvement in many of these cases was attributable to the treatment which they had been under prior to the trial of the oil. The meagerness of Dr. Bennett's details precludes us from instituting fairly such an enquiry. All that we can do therefore, is to inform the reader that vomicæ and caverns of the lungs have been, according to the author, cured by the ol. jecoris aselli. We ought to have quoted ere this, Dr. Bennett's theory of the *modus operandi* of this oil, which is as follows:

"The *modus operandi* of the oil may be said to consist in stimulating the lymphatic glands and vessels, and by these means increasing the activity of the capillary system. By its action on the former the process of assimilation is facilitated, and the appetite increased. The quality of the blood is thus improved, and so lastly the different organs and structures of the body become better nourished, and receive more *turgor vitalis*." (pp. 50-1.)

We shall conclude with quoting directions as to the cautions to be observed during the administration of the oil; and we cannot help, at the same time, expressing our fears that the oleum jecoris aselli will prove far from equal to the expectations which Dr. Bennett's treatise is likely to excite in its favour.

"1. *The diagnosis,* which must be correct, not only as regards the disease, but as regards its form.

"2. *The constitution.* The flaccid and phlegmatic bear it best, and the plethoric worst. In scrofula with torpidity it is directly indicated. If irritation be present, however, its employment requires management and great care.

"3. *The contra-indications.* These are plethora, a disposition to inflammation, profuse menstrual or hemorrhoidal discharges, total loss of appetite, nausea and vomiting, pain in the abdomen, and diarrhœa dependent on disease in the intestinal walls.

"4. *The time of administration.* It should not be given in the morning fasting, and its employment is not advisable during epidemics of dysentery or diarrhœa.

"5. *The quality of the oil.* The best is the clear brown or reddish variety, next in power is the yellow, and the least beneficial is the white. A sample of the oil employed should always be analysed, in order to determine whether it contain iodine as a constituent.

"6. *The dose.* In adults it should be gradually increased to a strong dose, such as six table-spoonfuls; in children a smaller dose, proportionate to the age.

"7. *The diet.* A fat animal diet supports the action of the oil. All substances abounding in starch are to be avoided.

"8. *The conjoined treatment.* Sometimes it is necessary to give aromatics and bitter tonics, and in numerous instances, especial medicines are required, according to the nature of the case. Not unfrequently a course of chalybeate waters has been found useful.

"9. *The duration of the treatment.* In every case it is necessary to give it a sufficient length of time. The chronic diseases in which it is beneficial can only yield slowly. In this respect the practitioner must be guided by its effects on the constitution.

"10 *The judicious suspension of its use.* 1st, Should it occasion derangement of the digestive organs, diarrhœa, increased flow of the menses, &c.; and, 2dly, after its employment has been continued five or six months. In this last case it should be suspended two or three weeks, and bitter or ferruginous tonics given, according to circumstances, to support the tone of the stomach." (pp. 174-5.)

